# Supplementary figures and images for: The decoy SNARE Tomosyn sets tonic versus phasic release properties and is required for homeostatic synaptic plasticity
Source: eLife. 2021 Oct 29;10:e72841. doi: 10.7554/eLife.72841 (PMC8612732; doi:10.7554/eLife.72841)

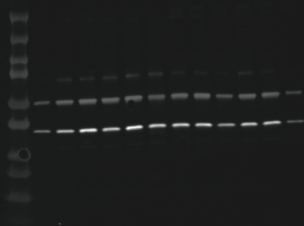

Supplement: Figure 1—source data 2. [file elife-72841-fig1-data2.pdf]

Tom<sup>NA1</sup>

Control

Analyzed lanes

Displayed image

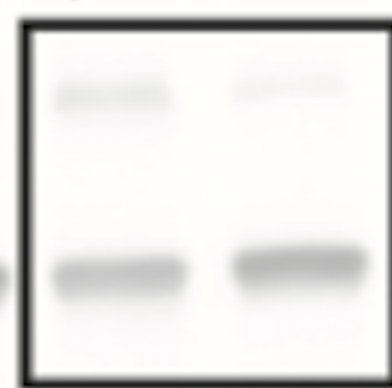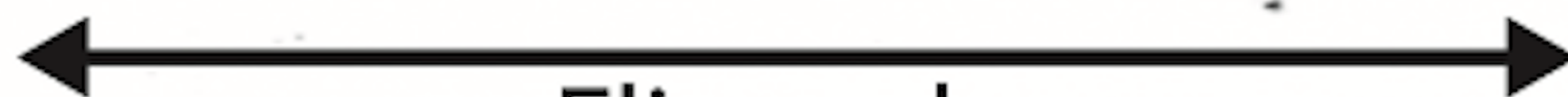

Flipped

Supplement: Figure 1—source data 3. [file elife-72841-fig1-data3.pdf]
